# Optimal Control with RdCVFL for Degenerating Photoreceptors

**DOI:** 10.1007/s11538-024-01256-6

**Published:** 2024-02-12

**Authors:** Kathryn Wifvat, Erika T. Camacho, Matthias Kawski, Thierry Léveillard, Stephen Wirkus

**Affiliations:** 1 School of Mathematical and Statistical Sciences, Arizona State University, Tempe, AZ 85287 USA; 2 Department of Mathematics, The University of Texas at San Antonio, San Antonio, TX 78249 USA; 3 School of Mathematical and Natural Sciences, Arizona State University, Glendale, AZ 85306 USA; 4 INSERM, U968, 75012 Paris, France; 5 UPMC Univ Paris 06, UMR_S 968, Institut de la Vision, Sorbonne Universités, 75012 Paris, France; 6 CNRS, UMR_7210, 75012 Paris, France

**Keywords:** Retinal degeneration, Retinitis pigmentosa, Optimal control, RdCVFL

## Abstract

Both the rod and cone photoreceptors, along with the retinal pigment epithelium have been experimentally and mathematically shown to work interdependently to maintain vision. Further, the theoredoxin-like rod-derived cone viability factor (RdCVF) and its long form (RdCVFL) have proven to increase photoreceptor survival in experimental results. Aerobic glycolysis is the primary source of energy production for photoreceptors and RdCVF accelerates the intake of glucose into the cones. RdCVFL helps mitigate the negative effects of reactive oxidative species and has shown promise in slowing the death of cones in mouse studies. However, this potential treatment and its effects have never been studied in mathematical models. In this work, we examine an optimal control with the treatment of RdCVFL. We mathematically illustrate the potential this treatment might have for treating degenerative retinal diseases such as retinitis pigmentosa, as well as compare this to the results of an updated control model with RdCVF.

## Introduction

### Background of Retinitis Pigmentosa and RdCVFL

Vision impairment is a serious problem that affects at least 2.2 billion people worldwide. Cataracts and glaucoma are the two most common causes of blindness in the world because developing countries rarely have access to the medical treatments needed to stop their progression and vision damage (Steinmetz et al. 2021). In contrast, the biggest contributors to vision loss in developed countries with no cure are due to photoreceptor degeneration, such as in age-related macular degeneration (AMD), untreated retinal detachment, and retinitis pigmentosa (RP) (Steinmetz et al. 2021). While a lot is known about these maladies, they still remain uncurable. The leading cause of inherited retinal degeneration that can cause complete loss of vision is RP, which affects about 1 in 4000 people (Besharse and Bok 2011; Shintani et al. 2009). RP is a heterogenous group of diseases characterized by rod photoreceptor death due to one of many different genetic mutations in the rods followed by the death of otherwise healthy cones. The evolving belief among experimentalists is that a combination of starvation and hyperoxia lead to this secondary cone death (Kanan et al. 2022; Léveillard and Sahel 2017; Petit et al. 2018). Recent experimental work has shown that the rod-derived cone viability factor (RdCVF), which is exclusively produced by the rods, increases glucose uptake by the cones; its long form (RdCVFL), produced by both rods and cones, has been shown to reduce photoreceptor degeneration by protecting against oxidative stress (Aït-Ali et al. 2015; Byrne et al. 2015; Elachouri et al. 2015; Léveillard et al. 2004). Thus, RdCVF and RdCVFL together provide a concrete link between starvation and hyperoxia once the rods have degenerated (Byrne et al. 2015; Elachouri et al. 2015; Léveillard and Sahel 2017). Mathematical work has confirmed the respective roles of RdCVF and RdCVFL as well (Aparicio et al. 2022; Camacho and Wirkus 2013; Camacho et al. 2014, 2016, 2016, 2019, 2021; Dobreva et al. 2022; Wifvat et al. 2021).

Both RdCVF and RdCVFL are encoded in the cDNA Nucleoredoxin-like (*Nxnl1*) gene (Chalmel et al. 2007; Léveillard et al. 2004). RdCVFL is a member of the thioredoxin family and includes a C-terminal extension that confers enzymatic activities (Brennan et al. 2010; Funato and Miki 2007) whereas RdCVF is truncated and has no enzymatic activity (Chalmel et al. 2007). However RdCVF is necessary to stabilize GLUT1 in the plasma membrane of photoreceptors, by binding to basigin-1 (Bsg1), in order to enhance glucose uptake (Aït-Ali et al. 2015). Thioredoxin is an important regulator of cellular redox homeostasis, which catalyzes the reduction of disulfide bonds and in humans it has been implicated in a wide variety of intracellular and extracellular redox regulations (Matsuo and Kimura-Yoshida 2013).

The rods produce the thioredoxin enzymatic protein RdCVFL as well as the trophic factor RdCVF through a process called alternative splicing. Cones on the other hand, only produce RdCVFL (Aït-Ali et al. 2015; Byrne et al. 2015). Therefore, both cones and rods express the RdCVFL protein and it is also helpful to both types of photoreceptor in protecting them from photo-oxidative damage (such as the oxidation of polyunsaturated fatty acids). In mice studies, it has been shown that RdCVFL reduces the effects of photoreceptor degeneration (Byrne et al. 2015). Just like any other protein from the thioredoxin family, the thiol oxidoreductase activity of RdCVFL depends on how high or reduced its oxidized status is. The thioredoxin reductases step in to reduce RdCVFL whenever it becomes oxidized (Léveillard et al. 2017). Usually produced from glucose in the pentose phosphate pathway, nicotinamide adenine dinucleotide phosphate (NADPH) is the cofactor of these reductases and thus most likely RdCVF provides a reducing power to RdCVFL (Dobreva et al. 2023; Léveillard et al. 2017). With a retinal disease such as RP, if the rods die then RdCVF is lost, which contributes to the secondary wave of cone death. It is speculated that part of the reason for the secondary cone death could be a result of oxidative damage due to the loss of RdCVFL from the rods (Elachouri et al. 2015) and thus it is necessary to study both RdCVF and RdCVFL to enhance our understanding of possible therapies that may prevent secondary cone deaths. However the first step towards investigating the joint effect of RdCVFL and RdCVF is to investigate the effects of each separately.

According to Mei et al. (2016), RdCVF is secreted by rods and protects cones by stimulating aerobic glycolysis when it interacts with glucose transporter 1 (GLUT1) and a complex containing basigin-1. The role of *Nxnl1* is studied in cones in this paper. Because RdCVFL is encoded by the *Nxnl1* gene, we consider *Nxnl1* to be a proxy for RdCVFL and thus the absence of RdCVFL is represented by the *Nxnl1*-/- mouse (a “knockout" mouse created to *not* have the *Nxnl1* gene (Elachouri et al. 2015)). The studies with mice confirm that damage produced by oxidative stress in cones can be reduced through the administration of RdCVFL to mice that have the genetic abnormality of a deletion of the *Nxnl1* gene (Elachouri et al. 2015). Further, RdCVFL is also shown to have cell-autonomous protection since cell viability is reduced when the RdCVFL expression is silenced in a cone-enriched culture (Elachouri et al. 2015). Thus both RdCVF and RdCVFL protect the cones in different ways and are each important and can both be incorporated into a treatment or therapy for RP.

RdCVFL protects rod and cone photoreceptors against photo-oxidative stress and damage as shown in mice studies (Byrne et al. 2015; Elachouri et al. 2015). The *rd10* mice are models of RP (e.g., see Phillips et al. 2010) and it is shown that RdCVFL reduces the oxidation of polyunsaturated fatty acids caused by the degeneration of photoreceptors (Byrne et al. 2015). Since RdCVFL includes a thioredoxin fold and RdCVF does not (however, it contains the thioredoxic catalytic site sequence CXXC), RdCVF has no thiol oxidoreductase activity (Léveillard et al. 2017). It is shown in Mei et al. (2016) that cones protect themselves against oxidative stress during aging as they produce RdCVFL. It has further been shown in mouse studies (Elachouri et al. 2015) and previous mathematical work (Wifvat et al. 2021) that RdCVFL can slow the death of cones by mitigating the negative effects of reactive oxidative species (ROS) (Clérin et al. 2011). Knowing how RdCVF and RdCVFL both interact with the cones sheds light on RP and suggests the potential for a double-therapeutic approach involving administration of both RdCVF and RdCVFL. Since potential theraputic effects of RdCVF have been studied previously, in this work, we will study the individual treatment with RdCVFL.

It has been experimentally shown previously by Mei et al. (2016), that RdCVFL causes a rescue effect for the cones. It has also been shown that RdCVFL is not expressed in the retinal pigment epithelium (RPE) of the *rd1* mouse (Mei et al. 2016; Léveillard and Sahel 2017, another mouse model of RP) but it is expressed in the photoreceptors of the eye. In lab studies with mice and a control group, RdCVFL was shown to reduce the damage done to cones by as much as 63% (Elachouri et al. 2015). The RdCVFL was subretinally injected after rods had degenerated and then the cone function was recorded. It was found that RdCVFL was able to protect cones from the secondary wave of death after rods had died by as much as 47% (Elachouri et al. 2015). One hypothesis of how this works is that after the rods die off and thus RdCVF is no longer being expressed, there is then less RdCVFL and a higher amount of oxidative stress which causes the cones to die (Léveillard and Sahel 2010). Thus, this protein shows a lot of promise for protecting cones even after rods have died, such as in later stages of RP.

A previous mathematical model looked at the administration of RdCVF and used optimal control theory to obtain the same rescue effect that was observed experimentally (Camacho et al. 2014). To the knowledge of the authors, this was the first use of optimal control utilizing treatment to try to prevent photoreceptor degeneration; a subsequent paper incorporating a different treatment via mesencephalic astrocyte-derived neurotrophic factor (MANF) again incorporated optimal control to reproduce the observed experimental data (Camacho et al. 2020). Optimal control has been used in a wide range of applications in mechanical engineering, economics, and a range of other disciplines (see, e.g., Athans and Falb 2013; Bryson 1975; Kirk 2004; Pontryagin 1987). In recent decades, optimal control in biological applications has become increasingly utilized (Lenhart and Workman 2007). Some applications to biological problems have included drug administration in chemotherapy, treatment strategies in epidemics, and population harvesting models to name a few (see, e.g., Lenhart and Workman (2007)).

This will be the first mathematical model of photoreceptors that includes optimal control treatment with RdCVFL to counteract photoreceptor degeneration. This information can potentially be used for developing treatments for trials. With optimal control, we maximize the amount of photoreceptors while minimizing the toxicity due to the potential treatments, to find the optimal amount of potential treatment for maintaining vision. Specifically, it is important to understand how the presence of RdCVFL impacts the cones and rods so that if a patient has a disease that affects their vision, potential treatments including RdCVFL can be used to help the patient maintain their night or peripheral vision as well as their central vision.

## ODE Model with RdCVFL Control and Derivations

The results presented in this manuscript build from the system of equations modeling the ecosystem of the rod cells, cone cells, and retinal pigmented epithelium (RPE):123which was analyzed in Wifvat et al. (2021); $R_n$ is the cumulative proportion of full length rod outer segments (OS), *C* is the cumulative proportion of full length cone outer segments, and *T* is the nutrients, including glucose, and other trophic factors mediated by the RPE. The parameters and their meanings are listed in Table 1 (Wifvat et al. 2021).

**Table 1:** Parameter descriptions and units; also, see Wifvat et al. (2021)

Parameter	Description	Units
$a_n$	Renewal and assoc. metabolism of rod OS mediated by T	$\frac{1}{{\text {day\,mM}}}$
$\beta _{{n}}$	Removal rate of nutrients from T by *R*	$\frac{1}{{\text {day\,(rod OS)}}}$
$\gamma$	Removal rate of nutrients from T by *C*	$\frac{1}{{\text {day\,(cone OS)}}}$
$\Gamma$	Total inflow rate into the T	$\frac{1}{{\text {day}}}$
$\kappa$	Limiting capacity of trophic factors	$\frac{1}{{\text {day\,mM}}}$
$\mu _n$	Metabolism assoc. w. energy cons & shedding of R	$\frac{{\text {mM}}}{{\text {day}}}$
$\mu _c$	Metabolism assoc. w. energy cons & shedding of C	$\frac{{\text {mM}}}{{\text {day}}}$
$K_{m{g}}$	Substrate concentration that gives half $V_{\max }$	mM
$B_1$	Quantification of reactive oxygen species for R	mM
$B_2$	Quantification of reactive oxygen species for C	mM
$V_{\max }$	Limiting value of the transport rate of glucose	$\frac{{\text {mM}}}{{\text {day}}}$
$\delta _n$	Per cell conc. of RdCVF synthesized by *R*	$\frac{{\text {mM}}}{{\text {rod OS}}}$
$\delta _r$	Per cell conc. of RdCVFL expressed by *R* helps R	$\frac{{\text {mM}}}{{\text {rod OS}}}$
$\delta _c$	Per cell conc. of RdCVFL expressed by *C* helps C	$\frac{{\text {mM}}}{{\text {cone OS}}}$
$\nu _2$	Conversion factor	$\frac{1}{{\text {mM}}^2}$
p	Rate of glucose uptake in C in absence of RdCVF	$\frac{{\text {mM}}}{{\text {day}}}$

The RdCVFL parameters, $\delta _c$ and $\delta _r$, are nonnegative; all other parameters are positive

We now introduce a control function to the model (1)–(3) to study the effects that RdCVFL treatment has on its equilibrium solutions. The system of equations modified to include the control $u \in [0,1]$ has effectiveness of treatment parameters $\sigma _1$ and $\sigma _2$ where the value for $\sigma _1$ is 192, which is $\delta _r R_{n0}$ rounded to the nearest whole number, and $\sigma _2$ is 234, $\delta _c C_0$ rounded to the nearest whole number where $R_n0$ and $C_0$ are the initial cumulative portion of full length of rod and cone outer segments, respectively.

4 $$\begin{aligned}{} & {} \frac{dR_n}{dt}=R_n Ta_n-\mu _n \left( \frac{ R_n}{B_1 + u\sigma _1}\right) \end{aligned}$$

5 $$\begin{aligned}{} & {} \frac{dC}{dt}=\nu _2\left( \frac{V_{\max }(\delta _nR_n)^2}{K_{mg}^2+(\delta _nR_n)^2}+p \right) CT-\mu _c \left( \frac{ C}{B_2 + u \sigma _2}\right) \end{aligned}$$

6 $$\begin{aligned}{} & {} \frac{dT}{dt}=T(\Gamma -\kappa T-\beta _n R_n-\gamma C). \end{aligned}$$

This system can be defined as the following for the rest of the paper,

7 $$\begin{aligned} \dot{x}= f(x,u), \end{aligned}$$

for convenience, with $x=(R_n,C,T)$. The treatment that administers RdCVFL is incorporated into the system in the second term of (4) and (5) as *u*, the control function of time *t*. This treatment is in the form of a subretinal injection of an adeno-associated virus (AAV) vector. Upon the injection of the AAV, the pumping mechanisms of the RPE cause the gradual and complete absorption of the fluid (Martin et al. 2002).

Standard methods in optimal control do not apply due to the nonlinear manner in which the control appears in the system. We thus linearize the system in order to show existence of a control for the linear system and then compare with the nonlinear results. Here the variables and parameters $R_n$, *C*, $B_1$ and $B_2$ are rearranged, only in the terms that include the control *u*, to set up the equations to be linearized in the next step:

8 $$\begin{aligned}{} & {} \frac{dR_n}{dt}=R_n Ta_n-\mu _n \frac{R_n}{B_1} \left( \frac{ 1}{1 + \frac{\sigma _1}{B_1} u}\right) \end{aligned}$$

9 $$\begin{aligned}{} & {} \frac{dC}{dt}=\nu _2\left( \frac{V_{\max }(\delta _nR_n)^2}{K_{mg}^2+(\delta _nR_n)^2}+p \right) CT-\mu _c \frac{C}{B_2}\left( \frac{1}{1 + \frac{\sigma _2}{B_2}u}\right) \end{aligned}$$

10 $$\begin{aligned}{} & {} \frac{dT}{dt}=T(\Gamma -\kappa T-\beta _n R_n-\gamma C). \end{aligned}$$

The linearization (first order Taylor’s approximation about $u=0$) is

11 $$\begin{aligned}{} & {} \frac{dR_n}{dt}=R_n Ta_n-\mu _n \frac{R_n}{B_1} \left( 1- \frac{\sigma _1}{B_1} u\right) \end{aligned}$$

12 $$\begin{aligned}{} & {} \frac{dC}{dt}=\nu _2\left( \frac{V_{\max }(\delta _nR_n)^2}{K_{mg}^2+(\delta _nR_n)^2}+p \right) CT-\mu _c \frac{C}{B_2}\left( 1- \frac{\sigma _2}{B_2}u\right) \end{aligned}$$

13 $$\begin{aligned}{} & {} \frac{dT}{dt}=T(\Gamma -\kappa T-\beta _n R_n-\gamma C). \end{aligned}$$

This system can be defined as the following for the rest of the paper:

14 $$\begin{aligned} \dot{x}= f_l(x,u), \end{aligned}$$

for convenience, with $x=(R_n,C,T)$.

Further, we will compare the control terms of the nonlinear (4)–(6) and linearized (11)–(13) models:

15 $$\begin{aligned}{} & {} F(u) = {\left\{ \begin{array}{ll} \mu _n \left( \frac{ R_n}{B_1 + u \sigma _1}\right) \\ \mu _c \left( \frac{ C}{B_2 + u \sigma _2}\right) \end{array}\right. } \end{aligned}$$

16 $$\begin{aligned}{} & {} {\tilde{F}}(u) = {\left\{ \begin{array}{ll} \mu _n \frac{R_n}{B_1} \left( 1- \frac{\sigma _1}{B_1} u \right) \\[.07in] \mu _c \frac{C}{B_2} \left( 1- \frac{\sigma _2}{B_2}u\right) \end{array}\right. } \end{aligned}$$

Most of the standard general theorems for the existence of optimal controls such as Filippov (1962), Neustadt (1963) and Fleming and Rishel (1975) require the set of velocities, in this case $F([u_{\min }, u_{\max }])$, to be convex.

**Fig. 1:**
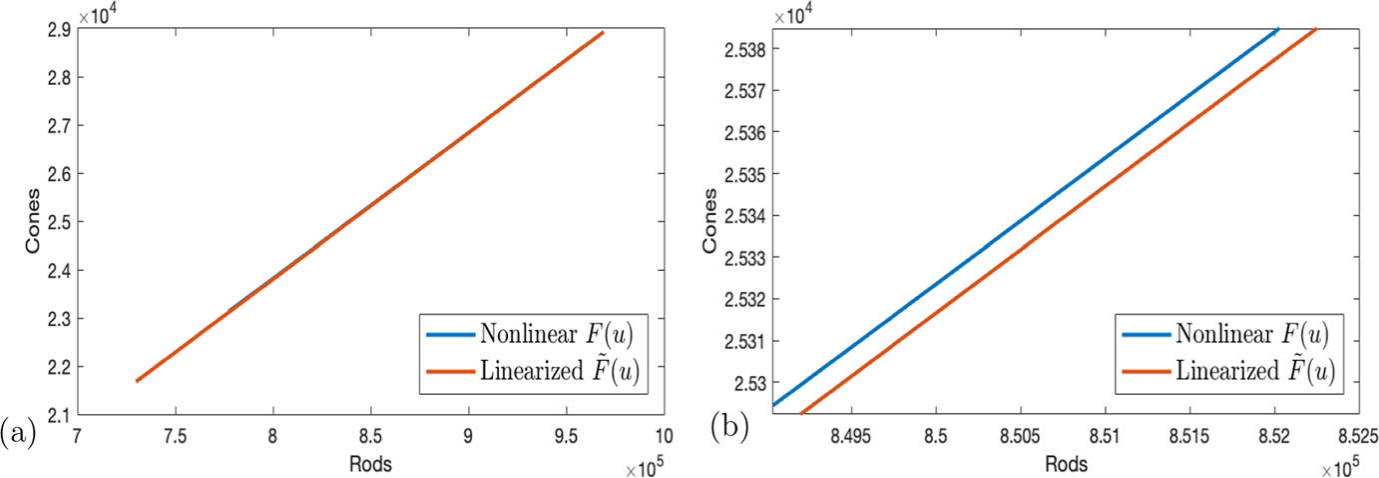
(Color figure online) **a** Left panel: the plots of *F*(*u*) and ${\tilde{F}}(u)$. Observe that these are practically indistinguishable at this scale. Right panel: Zoomed in plots of *F*(*u*) and ${\tilde{F}}(u)$

In our system (4)–(6), the controls enter nonlinearly, which is the most accurate biological model; however, we cannot ensure existence of optimal controls. Fortunately, due to the magnitude of the parameters $\frac{\sigma _i}{B_i}$ for $i=1, 2$ and the size of the control *u*, the linearization ${\tilde{F}}(u)$ of *F*(*u*) about 0 is extremely close to the original system; see Fig. 1. Note that the linearization ${\tilde{F}}(u)$ about $\frac{u_{\min }+u_{\max }}{2}$ would be even closer. However for easier readability, we choose to use the linearization about 0, which is already very close.

**Table 2:** Percent error for $R_n$ and *C* for *F*(*u*) and ${\tilde{F}}(u)$

	$R_n$	*C*
$F(u_{\min })$	7.77e5	2.31e4
${\tilde{F}}(u_{\min })$	7.30e5	2.17e4
% Error	6.09%	6.29%
$F(u_{\max })$	9.433e5	2.814e4
${\tilde{F}}(u_{\max })$	9.425e5	2.812e4
% Error	0.075%	0.078%

Percent error is taken separately for rods and cones, because in healthy conditions, they are an order of magnitude apart. Percent errors are taken at the endpoints; see Fig. 1a, b

Because the rods and cones are an order of magnitude apart in healthy conditions with rods to cones 30:1 in mice and 20:1 in humans, we do not use the Euclidean distance but instead report the errors for rods and cones separately. These distances are less than 10% error and less than 1% error respectively (Table 2). Because the control terms are of the form $\frac{A}{B+C u}$, the derivatives don’t change sign between $u_{\min }$ and $u_{\max }$ and so the maximum distance is at the endpoints. This shows that the control found for the nonlinear system must be very similar to the optimal control that exists. And, in the results that follow, it is shown that the optimal control for the linearized system is indeed very similar.

## Optimal Control

### Objective Functional

Next the objective functional is defined for both the nonlinear (4)–(6) and linearized (11)–(13) control models. This optimal control problem is formulated to take into account the administration of RdCVFL to the cones because of the fact that the secondary death of cones is a result of being exposed to oxidative stress and the inclusion of RdCVFL protects from that stress. While using the cumulative portion of full length of cone outer segments, to maximize cone longevity the objective functional also takes care to minimize the dosage of RdCVFL to the cones. The control is denoted by the function $u\!\!:[0,t_f] \rightarrow {[0,1]}$ and is a representation of the percentage of RdCVFL which is administered to assist in cone survival over the time period $[0,t_f]$. This optimal control problem will be compared to experimental results from Elachouri et al. (2015) and thus the dosage *u* will be considered as being administered over the course of ten days. The objective functional is defined as

17 $$\begin{aligned} I(u)=\int _{0}^{t_f}\left( \frac{\epsilon }{2}(u(t))^2-(\alpha _1 R_n + \alpha _2 C)\right) {d}t \end{aligned}$$

where the importance of minimizing *u* is represented as the weight $\epsilon$ and the set of controls is defined as

18 $$\begin{aligned} U=\{u|u \ \text {is measurable,} \ u_{\min } \le u(t) \le u_{\max }, \ t \in [0,t_f]\} \end{aligned}$$

and this set is closed. The set *U* is an interval, and hence it is convex. Note that $u_{\min }=0, u_{\max }=0.9$ to allow for flexibility in efficiency.

### Existence of Unique Global Solutions for the Nonlinear System

#### Theorem 1

For every fixed measurable control $u: [0, t_f] \mapsto [u_{\min }, u_{\max }]$ and every initial condition ($R_{n0}, C_0, T_0$) in ${\bar{\Omega }} = \{(R,C,T):R\ge 0, C\ge 0, T\ge 0\}$, the system defined in (4)–(6) has a locally unique and bounded solution defined on $[0, t_f]$.

#### Proof

By hypothesis, the controls are bounded and measurable functions of time *t*. For every fixed $(R_n, C, T)$ and fixed control $u(\cdot )$, the right hand side *f* is a measurable function of time, as a composition of a measurable function and a continuous function in the “good” order (Royden and Fitzpatrick 1988). For each fixed *t*, the vector field $f(\cdot ,u(t))$ is a rational function of $(R_n,C,T)$ without any singularities in the closed first octant, and hence is locally Lipschitz continuous on this set. Using the boundedness of the controls, one can show analogously to Theorem 6 (see “Appendix A”) that solutions for this *u* stay bounded and are thus defined for all positive times *t* less than or equal to $t_f$. The calculations are the same, except in (39), the term $\delta _r R_n$ is replaced with $u_{\max } \sigma _1$, and $\delta _c C$ is replaced with $u_{\max } \sigma _2$. By the Carathéodory theorem (Coddington and Levinson 1955 Theorem 1.1), there exists a unique solution on the interval $[0, t_f]$ for this control *u*. $\square$

### Existence of Unique Global Solutions for the Linearized System

#### Theorem 2

For every fixed measurable control $u: [0, t_f] \mapsto [u_{\min }, u_{\max }]$ and every initial condition ($R_{n0}, C_0, T_0$) in ${\bar{\Omega }} = \{(R,C,T):R\ge 0, C\ge 0, T\ge 0\}$, the system defined in (11)–(13) has a locally unique and bounded solution defined on $[0, t_f]$.

#### Proof

By hypothesis, the controls are bounded and measurable functions of time *t*. For every fixed $(R_n, C, T)$ and fixed control $u(\cdot )$, the right hand side $f_l$ is a measurable function of time. For each fixed *t*, the vector field $f_l(\cdot ,u(t))$ is a rational function of $(R_n,C,T)$ without any singularities in the closed first octant, and hence is locally Lipschitz continuous on this set. Using the boundedness of the controls, one can show analogously to Theorem 6 that solutions for this *u* stay bounded and are thus defined for all positive times *t* less than or equal to $t_f$. The calculations are the same, except in (39) the term $\frac{R_n}{B_1+\delta _r R_n}$ is replaced with $\frac{R_n}{B_1}\left( 1-\frac{\sigma _1}{B_1} u \right)$, and $\frac{C}{B_2+\delta _c C}$ is replaced with $\frac{C}{B_2}\left( 1-\frac{\sigma _2}{B_2} u \right)$. By the Carathéodory theorem (Coddington and Levinson 1955 Theorem 1.1), there exists a unique solution on the interval $[0, t_f]$ for this control *u*. $\square$

### Existence of Optimal Control for the Linearized System

Because most standard general theorems for the existence of optimal controls require the set of velocities to be convex, we only show the existence of optimal controls for the system (11)–(13) where the controls enter linearly.

We will verify that the optimal control problem (11)–(13) together with the objective functional (17) satisfies the conditions of Theorem 2.2 from Lenhart and Workman (2007) for the basic problem (1.2) (p. 7 of the same reference). To keep this manuscript self-contained, we paraphrase the statement of that theorem using our terminology and notation, such as noting that the dynamics and cost functional do not explicitly depend on time.

The following theorem refers to the problem of minimizing a cost functional (equation (1.2) in Lenhart and Workman (2007)) $J(u)=\int _0^{t_1}\ell (x(t),u(t))\,dt$ subject to $x'(t)=f(x(t),u(t))$, $x(0)=x_0$ with *f* and $\ell$ continuously differentiable.

#### Theorem 3

(Lenhart and Workman, paraphrased and customized) Let the set of controls be Lebesgue measurable on $0\le t\le t_1$ with values in ${\mathbb {R}}$. Suppose that *f*(*x*, *u*) is convex in *u*, and there exists constants $C_4$ and $C_1,\,C_2,\,C_3>0$, and $\beta >1$ such that for all $t\in [0,t_1]$, and all $x,\,x_1,u\in {\mathbb {R}}$

19 $$\begin{aligned} \begin{array}{cl} \mathrm {(i)} &{} f(x,u) = \alpha (x)+\beta (x)u,\\ \mathrm {(ii)} &{} |f(x,u)| \le C_1(1+|x|+|u|),\\ \mathrm {(iii)} &{} |f(x_1,u)-f(x,u)| \le C_2(|x_1-x|)(1+|u|),\; \text{ and } \\ \mathrm {(iv)} &{}|\ell (x,u)| \ge C_3|u|^\beta - C_4. \end{array} \end{aligned}$$

Then there exists an optimal control $u^*$ minimizing *J*(*u*) with $J(u^*)$ finite.

To verify that this theorem applies to our system, first note that with Corollary 1 and Remark 1 in the appendix, we may restrict our considerations to a compact, forward invariant *simplex* $S'$ in the first octant. On this set, the dynamics *f*(*x*, *u*) is affine in the control *u* and rational in the state $x=(R,C,T)$ and without singularities in the first quadrant, and thus (i) and (ii) are satisfied. Since *f* is continuously differentiable it is Lipschitz continuous on the compact set $S'$ with Lipschitz constant independent of the control, and thus (iii) is satisfied. The Lagrangian in the functional (17) clearly is of the form (iv) with $\beta =2$ and with finite $C_4$ since the term $(\alpha _1 R_n + \alpha _2 C)$ is linear in the state variables and thus bounded on the domain $S'$ of interest.

### Optimality Conditions

There are first-order necessary optimality conditions which must be met for the optimal control problem that will be outlined in this section. The optimal control problem, subject to the nonlinear state equations (4)–(6) with objective functional *I*(*u*) must meet the following conditions in order for the optimal control $u^*(t)$ to be characterized.

The previous equations will be rewritten as the following maximization problem for convenience:

20 $$\begin{aligned} \max _u J(u) \ \text {subject to}\ \dot{x} = f(x,u) \end{aligned}$$

where

21 $$\begin{aligned} J(u)=-I(u)=\int _0^{t_f} \left( \alpha _1 R_n + \alpha _2 C-\frac{\epsilon }{2}u^2\right) dt. \end{aligned}$$

Thus an optimal control $u^*$ must be as follows:

22 $$\begin{aligned} J(u^*)=\max _{u \in U} J(u)\ \text {subject to} \ \dot{x}=f(x,u^*). \end{aligned}$$

Then continuing to Pontryagin’s Maximum Principle, the Hamiltonian is first defined:

23 $$\begin{aligned} H(x,\lambda , u)=\alpha _1 R_n + \alpha _2 C-\frac{\epsilon }{2}u^2+\lambda ^Tf(x,u) \end{aligned}$$

where next, $\lambda$ is defined as the vector of adjoint variables, which are represented as $\lambda \equiv \lambda (t) = (\lambda _1(t),\lambda _2(t),\lambda _3(t))^T$. Then we let $H\equiv H(x,\lambda , u)$ and $\lambda _i\equiv \lambda _i(t)$ with $i=1,\dots ,3$ for convenience.

*Nonlinear System*

Even though an optimal control is not guaranteed to exist, we proceed with the standard technique for finding one and then compare with the linear version. The optimal control that we find given the standard procedure will be referred to as the putative optimal control.

Using the nonlinear system (4)–(6) and the objective function, the Hamiltonian is defined as follows:

24 $$\begin{aligned} \begin{aligned} H&= \alpha _1 R_n + \alpha _2 C-\frac{\epsilon }{2}u^2\\&\quad +\lambda _1 \left( R_n Ta_n-\mu _n \left( \frac{R_n}{B_1 + u \sigma _1 }\right) \right) \\&\quad +\lambda _2 \left( \nu _2\left( \frac{V_{\max }(\delta _nR_n)^2}{K_{mg}^2+(\delta _nR_n)^2}+p \right) CT-\mu _c \left( \frac{ C}{B_2 + u \sigma _2 }\right) \right) \\&\quad +\lambda _3 \left( T\left( \Gamma - kT-\beta _nR_n-\gamma C\right) \right) \end{aligned} \end{aligned}$$

#### Theorem 4

Pontryagin’s Maximum Principle: If u* and $x^*=(R_n^*, C^*, T^*)$ are optimal for problem (22) then there exist absolutely continuous and piecewise differentiable adjoint functions $\lambda _i:[0,t_f]\rightarrow {{\mathbb {R}}}$ for $i=1,2,3,4$ such that

25 $$\begin{aligned}{} & {} \begin{aligned} \frac{\partial \lambda _1}{\partial t}&= - \frac{\partial H}{\partial R_n}(x^*, u^*) = -\alpha _1 -\lambda _1 \left( T^* a_n - \frac{\mu _n}{B_1+ u \sigma _1 } \right) +\lambda _3 \beta _n T^* \\&\quad -\lambda _2 \left( \nu _2 \left( \frac{2K_{mg}^2V_{\max }\delta _n^2R_n^*}{(K_{mg}^2+(\delta _nR_n^*)^2)^2} \right) C^*T^* \right) \end{aligned} \end{aligned}$$

26 $$\begin{aligned}{} & {} \begin{aligned} \frac{\partial \lambda _2}{\partial t}&= - \frac{\partial H}{\partial C}(x^*, u^*) = -\alpha _2 +\lambda _3 \gamma T^* \\&\quad - \lambda _2\left( \nu _2 \left( \frac{V_{\max } (\delta _n R_n^*)^2}{K_{mg}^2+(\delta _nR_n^*)^2}+p \right) T^* -\frac{\mu _c}{B_2+u \sigma _2 }\right) \end{aligned} \end{aligned}$$

27 $$\begin{aligned}{} & {} \begin{aligned} \frac{\partial \lambda _3}{\partial t}&= - \frac{\partial H}{\partial T}(x^*, u^*) = -\lambda _1 R_n^* a_n -\lambda _3(\Gamma -2\kappa T^*-\beta _n R_n^*-\gamma C^*)\\&\quad -\lambda _2 \left( \nu _2 \left( \frac{V_{\max } (\delta _n R_n^*)^2}{K_{mg}^2+(\delta _nR_n^*)^2}+p \right) C^*\right) . \end{aligned} \end{aligned}$$

The costate $\lambda$ satisfies the transversality conditions being

$$\begin{aligned} \lambda _i(t_f)=0, \ \text {for} \ i=1,\dotsc , 3. \end{aligned}$$

For all $t \in [t_0, t_f]$, the optimal control $u^*(t)$ maximizes the Hamiltonian, i.e. $\forall \ u \in [u_{\min }, u_{\max }], \ H(x^*(t), \lambda (t), u^*(t)) \ge H(x^*(t), \lambda (t), u)$.

*Linearized System*

For the linearized system (11)–(13), the Hamiltonian is defined as follows:

28 $$\begin{aligned} \begin{aligned} H&= \alpha _1 R_n + \alpha _2 C-\frac{\epsilon }{2}u^2\\&\quad +\lambda _1 \left( R_n Ta_n-\mu _n \frac{R_n}{B_1} \left( 1- \frac{\sigma _1}{B_1} u\right) \right) \\&\quad +\lambda _2 \left( \nu _2\left( \frac{V_{\max }(\delta _nR_n)^2}{K_{mg}^2+(\delta _nR_n)^2}+p \right) CT-\mu _c \frac{C}{B_2} \left( 1- \frac{\sigma _2}{B_2} u\right) \right) \\&\quad +\lambda _3 \left( T\left( \Gamma - kT-\beta _nR_n-\gamma C\right) \right) \end{aligned} \end{aligned}$$

#### Theorem 5

Pontryagin’s Maximum Principle: If u* and $x^*=(R_n^*, C^*, T^*)$ are optimal for problem (22) then there exist absolutely continuous and piecewise differentiable adjoint functions $\lambda _i:[0,t_f]\rightarrow {{\mathbb {R}}}$ for $i=1,2,3,4$ such that

29 $$\begin{aligned}{} & {} \begin{aligned} \frac{\partial \lambda _1}{\partial t}&= - \frac{\partial H}{\partial R_n}(x^*, u^*) = -\alpha _1 -\lambda _1 \left( T^* a_n - \frac{\mu _n}{B_1}\left( 1 - \frac{\sigma _1 u}{B_1} \right) \right) +\lambda _3 \beta _n T^*\\&\quad -\lambda _2 \left( \nu _2 \left( \frac{2K_{mg}^2V_{\max }\delta _n^2R_n^*}{(K_{mg}^2+(\delta _nR_n^*)^2)^2} \right) C^*T^* \right) \end{aligned} \end{aligned}$$

30 $$\begin{aligned}{} & {} \begin{aligned} \frac{\partial \lambda _2}{\partial t}&= - \frac{\partial H}{\partial C}(x^*, u^*) = -\alpha _2 +\lambda _3 \gamma T^*\\&\quad - \lambda _2\left( \nu _2 \left( \frac{V_{\max } (\delta _n R_n^*)^2}{K_{mg}^2+(\delta _nR_n^*)^2}+p \right) T^*-\frac{\mu _c}{B_2} \left( 1 - \frac{\sigma _2 u}{B_2}\right) \right) \end{aligned} \end{aligned}$$

31 $$\begin{aligned}{} & {} \begin{aligned} \frac{\partial \lambda _3}{\partial t}&= - \frac{\partial H}{\partial T}(x^*, u^*) = -\lambda _1 R_n^* a_n -\lambda _3(\Gamma -2\kappa T^*-\beta _n R_n^*-\gamma C^*)\\&\quad -\lambda _2 \left( \nu _2 \left( \frac{V_{\max } (\delta _n R_n^*)^2}{K_{mg}^2+(\delta _nR_n^*)^2}+p \right) C^*\right) . \end{aligned} \end{aligned}$$

The costate $\lambda$ satisfies the transversality conditions being

$$\begin{aligned} \lambda _i(t_f)=0, \ \text {for} \ i=1,\dotsc , 3. \end{aligned}$$

For all $t \in [t_0, t_f]$, the optimal control $u^*(t)$ maximizes the Hamiltonian, i.e. $\forall \ u \in [u_{\min }, u_{\max }], \ H(x^*(t), \lambda (t), u^*(t)) \ge H(x^*(t), \lambda (t), u)$.

### Description of the Discretized Variables

In this section an approximate solution to the system of equations (4)–(6), $\textbf{x}$, is described. The following nodes describe how the time interval $[0,t_f]$ is discretized into *N* subintervals that are equally spaced with

$$\begin{aligned} h=t_{i+1}-t_i,\ \text {for} \ i=0,\dotsc ,N-1, \end{aligned}$$

and

32 $$\begin{aligned} 0=t_0<t_1< \cdots < t_N=t_f. \end{aligned}$$

Next, the discretized vector $\textbf{x}$ at time $t_k$ is defined as

$$\begin{aligned} \mathbf {x_k}= (R_n(t_k),C(t_k),T(t_k))^T. \end{aligned}$$

It follows similarly that for $k=0,\ldots ,N$, $u_k=u(t_k)$ and for all time steps $t_k$ with $k=0,1,2,\ldots N$, all discretized values are contained in the vector $\textbf{u}=[u_0,u_1,u_2,\dotsc ,u_N]$.

Next, an approximation to the solution of the system of equations (4)–(6) for the state variable $\textbf{x}$ is calculated given initial conditions $x_0 \in R^3$ with initial $\textbf{u}$ over the time interval $[0,t_f]$, by implementing the fourth-order Runge–Kutta method forward in time with $k=0,\dotsc ,N$. The forward Runge–Kutta method is as follows:

$$\begin{aligned} \textbf{k}_1= & {} \textbf{f}\left( t_k,\textbf{x}_k,u_k\right) \\ \textbf{k}_2= & {} \textbf{f}\left( t_k+\frac{h}{2},\textbf{x}_k+\frac{h}{2}\textbf{k}_1,\frac{1}{2}(u_k+u_{k+1})\right) \\ \textbf{k}_3= & {} \textbf{f}\left( t_k+\frac{h}{2},\textbf{x}_k+\frac{h}{2}\textbf{k}_2,\frac{1}{2}(u_k+u_{k+1})\right) \\ \textbf{k}_4= & {} \textbf{f}\left( t_{k+1},\textbf{x}_k+\textbf{k}_3,u_{k+1}\right) \\ \textbf{x}_{k+1}= & {} \textbf{x}_k+\frac{h}{6}\left( \textbf{k}_1+2\textbf{k}_2+2\textbf{k}_3+\textbf{k}_4\right) . \end{aligned}$$

It is important to note that there are several different ways to approximate the value of $u_k$ in calculating $\textbf{k}_2$ and $\textbf{k}_3$. In this algorithm, we replace $u_k$ with the average, $\frac{1}{2}(u_k+u_{k+1})$, because this has been shown to be sufficient (Lenhart and Workman 2007).

Next, the Runge–Kutta method is implemented backward in time to numerically solve the adjoint system and compute the solution $\lambda$. The definitions of *h* and discretization of the time variable $t \in [0,t_f]$ remains the same as stated previously. Define the adjoint vector $\mathbf {\lambda }_k \in {\mathbb {R}}^3$, for which the values of the adjoint variables at each discrete time $t_k$ are the components, as $\mathbf {\lambda }_k=(\lambda _1(t_k),\lambda _2(t_k),\lambda _3(t_k))^T$. The initial iterate is set to $\mathbf {\lambda }_N=0$ in order to enforce the transversality condition. Then, the backward solve Runge-Kutta method is as follows for $k=N,N-1, \dotsc , 1$:

$$\begin{aligned} \textbf{k}_1= & {} \textbf{g}\left( t_k,\mathbf {\lambda }_k,\textbf{x}_k,u_k\right) \\ \textbf{k}_2= & {} \textbf{g}\left( t_k-\frac{h}{2},\mathbf {\lambda }_k-\frac{h}{2}\textbf{k}_1, \frac{1}{2}(\textbf{x}_k+\textbf{x}_{k-1}),\frac{1}{2}(u_k+u_{k-1})\right) \\ \textbf{k}_3= & {} \textbf{g}\left( t_k-\frac{h}{2},\mathbf {\lambda }_k-\frac{h}{2}\textbf{k}_2, \frac{1}{2}(\textbf{x}_k+\textbf{x}_{k-1}),\frac{1}{2}(u_k+u_{k-1})\right) \\ \textbf{k}_4= & {} \textbf{g}\left( t_{k-1},\mathbf {\lambda }_k-\textbf{k}_3,\textbf{x}_{k-1},u_{k-1}\right) \\ \mathbf {\lambda }_{k-1}= & {} \mathbf {\lambda }_k-\frac{h}{6}\left( \textbf{k}_1+2\textbf{k}_2+2\textbf{k}_3+\textbf{k}_4\right) . \end{aligned}$$

### Updating the Control Variable

*Nonlinear System*

Next, the characterization of the optimal control is derived using Pontryagin’s Maximum Principle. By this principle, the Hamiltonian must satisfy

33 $$\begin{aligned} \frac{\partial H}{\partial u}=0=-\epsilon u + \frac{\lambda _1 \mu _n \sigma _1 R_n}{(B_1 + u \sigma _1 )^2}+\frac{\lambda _2 \mu _c \sigma _2 C}{(B_2 + u \sigma _2 )^2} \end{aligned}$$

at optimality. Rearranging gives

34 $$\begin{aligned} \epsilon u = \frac{\lambda _1 \mu _n \sigma _1 R_n}{(B_1 + u \sigma _1 )^2}+\frac{\lambda _2 \mu _c \sigma _2 C}{(B_2 + u \sigma _2 )^2},\end{aligned}$$

where we observe that since all the parameters in (34) are positive, there will be only one intersection of $\epsilon u$ with the right-hand-side, and this intersection will occur in the first quadrant. However, solving for *u*, and only taking the unique real root of (33), the analytic solution is too cumbersome to enter into MATLAB, and so it is solved for using a root finding method. Then, *u* becomes the average of the new value *u* and the old value for *u*, for each time step *k*.

Then, a new vector of control variables, $\textbf{u}=[u_0,u_1,u_2,\dotsc , u_N]$, is formed to test convergence once all the values over the discretized time interval $[0,t_f]$ have been calculated.

*Linearized System*

Now for the linearized system the characterization of the optimal control is derived using Pontryagin’s Maximum Principle. For this system, the Hamiltonian must satisfy

35 $$\begin{aligned} \frac{\partial H}{\partial u}=0=-\epsilon u + \frac{\lambda _1 \mu _n \sigma _1 R_n}{B_1^2}+\frac{\lambda _2 \mu _c \sigma _2 C}{B_2^2} \end{aligned}$$

at optimality.

Solving for *u*, we get

36 $$\begin{aligned} u=\frac{\lambda _2 \mu _c C \sigma _2 B_1^2 + \lambda _1 \mu _n R_n \sigma _1 B_2^2}{B_1^2 B_2^2 \epsilon }. \end{aligned}$$

Then, *u* becomes the average of the new value *u* and the old value for *u*, for each time step *k*. After that, a new vector of control variables, $\textbf{u}=[u_0,u_1,u_2,\dotsc , u_N]$, is formed to test convergence once all the values over the discretized time interval $[0,t_f]$ have been calculated. We observe that Taylor expanding the nonlinear terms in the characterization of *u* for the nonlinear equations and keeping linear terms, (33), gives the exact same characterization as (36).

### The Forward-Backward Sweep Method (FBSM)

#### FBSM Algorithm

The numerical approximation of the optimality condition can be summarized by the following algorithm (Lenhart and Workman 2007). Assume that the initial condition is given as $\textbf{x}(t_0)$, over the uniformly discretized time interval $[0,t_f]$ with mesh size *h* and stopping criteria given by $\delta$.

1. Initialize $$\textbf{u}=0$$.
2. Approximate solution $\textbf{x}$ to (ODE) forward in time *t* over time interval $[0,t_f]$ using $\textbf{u}$ and $\textbf{x}(t_0)$.
3. Approximate solution $\mathbf {\lambda }$ to (ADJ) backward in time *t* over $[0,t_f]$ using $\textbf{u}$, $\textbf{x}(t_0)$ and $\mathbf {\lambda }(t_f)=0$.
4. Update $\textbf{u}$ using the characterization formula, equation (34) for the nonlinear system or (36) for the linear system.
5. Test for convergence. If convergence is not met, return to 2. and repeat steps 2–5 using the updated values for $\textbf{u}$ and $\textbf{x}(t_0)$.

**Fig. 2:**
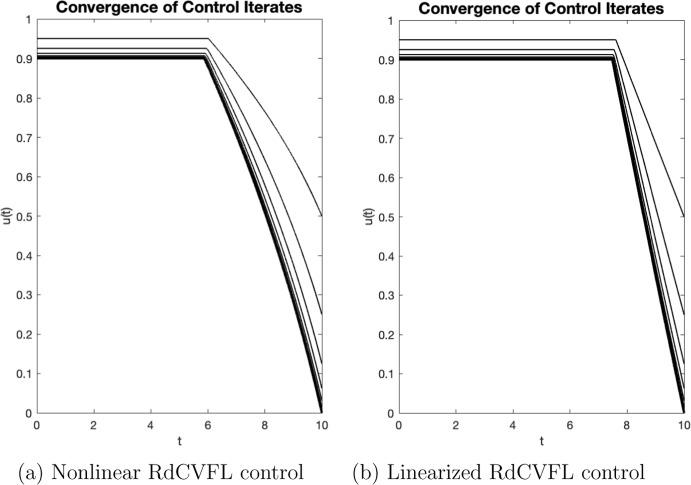
Plots of the control iterates for the RdCVFL control models with $\epsilon =7\text {e}5$. **a** Plot of the control estimates for the nonlinear model with control (4)–(6). Recall that for this case, we do not have proof of existence of optimal control. However, for the sake of comparison and biological relevance, we follow the same methods to find $u^*$, which we call the putative optimal control in this instance. The putative optimal control $u^*$ is depicted by the bold line. Observe that the control iterates converge to $u^*$, which is achieved in 12 iterations. **b** Plot of the control estimates for the linearized model with control (11)–(13). In this case, we do have a mathematical proof of existence for the optimal control. The optimal control $u^*$ is depicted by the bold line. Observe that the control iterates converge to $u^*$, which is achieved in 12 iterations

Each single iteration is defined as each single completion of steps 2–5. When approximating solutions numerically for optimal control problems, this algorithm is the standard procedure; however, it has limitations (Lenhart and Workman 2007). It is also important to note that the solutions do not always converge following FBSM (McAsey et al. 2012). General assumptions under which this algorithm converges are defined in McAsey et al. (2012), and the authors also analyze an example of an optimal control problem for which there is a known analytical solution (Lenhart and Workman 2007), yet for which after 1000 iterations there is still no convergence. The magnitude of the coefficient of a term in the objective functional to the length of the time interval is what this nonconvergence is attributed to by the authors. Figure 2 shows the convergence for nonlinear and linearized models.

#### Stopping Criteria for the FBSM

The following discretization of $\textbf{u}\equiv \textbf{u}(t)\in {\mathbb {R}}^{N+1}$ over the discretization of time $[0,t_f]$ holds the estimated values for the Forward-Backward Sweep Method (FBSM) for each current iteration:

$$\begin{aligned} \textbf{u}=(u(t_0),u(t_1),u(t_2),\dotsc , u(t_f)). \end{aligned}$$

It follows that the estimated values of *u* from the previous iteration of FBSM is defined as $\textbf{u}_{old}\in {\mathbb {R}}^{N+1}$. See, for example, Lenhart and Workman (2007).

Once the estimated values of *u* are calculated at each iteration, a stopping criteria must be met for FBSM using relative errors for the control, state variables, and adjoint variables. This implementation is now demonstrated using a given tolerance $\delta$ and the values for the control *u* as an example that covers the other variables listed in general. The relative error $\delta$ is now defined in terms of the control *u* and the $\ell _1$ vector norm:

$$\begin{aligned} \frac{||\textbf{u}-\textbf{u}_{old}||}{||\textbf{u}||}\le \delta , \end{aligned}$$

which can be rewritten as the following in order to take into account the fact that the control variable may have the value 0:

37 $$\begin{aligned} \delta ||\textbf{u}||-||\textbf{u}-\textbf{u}_{old}|| \ge 0. \end{aligned}$$

Next, $\beta _1$ is defined following the work done by Lenhart and Workman (2007):

$$\begin{aligned} \beta _1= \delta ||\textbf{u}||-||\textbf{u}-\textbf{u}_{old}||. \end{aligned}$$

Now, for each current iteration of the FBSM method, the relative errors for each of the state variables can be rewritten. The state variables defined as $\textbf{X}=(\mathbf {X_1},\mathbf {X_2},\mathbf {X_3})^T \in {\mathbb {R}}^{3 \times (N+1)}$. These relative errors which are obtained from the current iteration are given in the matrix:

$$\begin{aligned} \textbf{X} = \left( \begin{array}{c} \mathbf {X_1}\\ \mathbf {X_2}\\ \mathbf {X_3} \end{array} \right) = \left( \begin{array}{c} x_1(t_0) \quad x_1(t_1) \quad x_1(t_2) \quad \cdots \quad x_1(t_f) \\ x_2(t_0) \quad x_2(t_1) \quad x_2(t_2) \quad \cdots \quad x_2(t_f) \\ x_3(t_0) \quad x_3(t_1) \quad x_3(t_2) \quad \cdots \quad x_3(t_f) \end{array} \right) \end{aligned}$$

For each previous iteration of FBSM, the estimated state variables are also stored, in a matrix that is constructed in the same fashion as $\textbf{X}$. This matrix is defined as $\textbf{X}_{old} \in {\mathbb {R}}^{3 \times (N+1)}$ with the difference being that the *i*th row is denoted by $\textbf{X}_{old_i}$ instead of $\textbf{X}_i$. This *i*th row is defined as follows for both of these matrices:

$$\begin{aligned} \beta _{i+1}= \delta ||\textbf{X}_i||-||\textbf{X}_i-\textbf{X}_{old_i}||, \ \ i=1,\dotsc , 3. \end{aligned}$$

Next, the adjoint variables, defined as $\mathbf {\lambda }_i, i=1,\dotsc , 3$, must be taken into consideration and the process must be repeated for them. The estimated values of adjoint variables are similarly calculated using the current iteration of FBSM, which are then stored in the matrix $\Lambda =(\Lambda _1,\Lambda _2,\Lambda _3)^T\in {\mathbb {R}}^{4 \times (N+1)}$. Once again, $\Lambda _{old} \in {\mathbb {R}}^{3 \times (N+1)}$ is a matrix created using the previously estimated values of FBSM from the last iteration, and it follows that

$$\begin{aligned} \beta _{i+1}= \delta ||\Lambda _i||-||\Lambda _i-\Lambda _{old_i}||, \ \ i=4,\dotsc , 6. \end{aligned}$$

Then, the following is obtained:

$$\begin{aligned} \{ \beta _1,\beta _2,\dotsc , \beta _7 \}. \end{aligned}$$

Next, for the stopping criteria to be met in order to obtain convergence, the following must hold:

$$\begin{aligned} \min \{ \beta _1,\beta _2,\dotsc , \beta _7 \}>0, \end{aligned}$$

and if this inequality holds, then convergence has been obtained and the process is halted. If the stopping criteria has not been met, then the process is repeated with an additional run through the FBSM method.

## Numerical Results of Control Applied to Rods and Cones with RdCVFL

**Fig. 3:**
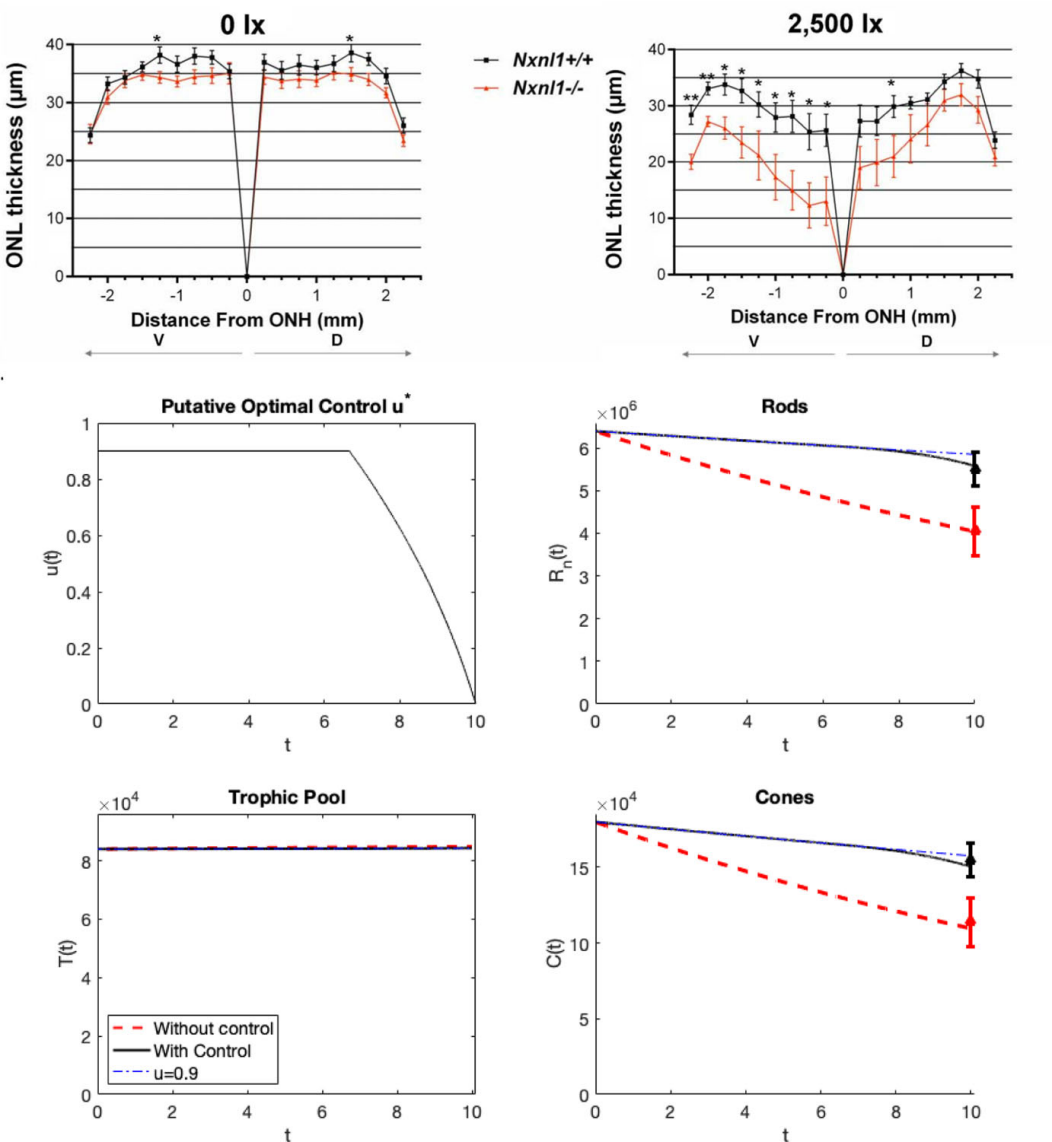
(Color figure online) The results of decay of both cones and rods, for the nonlinear RdCVFL control model (4)–(6). The initial conditions are $$R_n= 6.4\text {e}6, C= 1.8\text {e}5, T=8\text {e}4$$, over the course of 10 days (reflected by the experimental results of Elachouri et al. (2015) for the $${Nxnl1} $$-/- mouse, presented in the top panel), without control compared to the results of adding control to the rods and cones. The data points at time $$t=10$$ for the rods and cones are from experimental results of Elachouri et al. (2015)

Our nonlinear model (4)–(6) is based on the experimental results from Elachouri et al. (2015) for the *Nxnl1*-/- mouse. While we don’t have a mathematical proof for existence of the optimal control, this experiment shows a decrease in rods and cones over the course of 10 days, with long term behavior approaching $E_2$, where all the rods and cones have died off while the nutrients remain. Table 3 gives the parameter values that fit the data and give $E_5$ stable, with $B_1$, $B_2$, $\delta _r$ and $\delta _c$ from (1)–(3) having altered values to reflect the case of mice that do not have RdCVFL and have experienced light damage, where we consider *Nxnl1* to be a proxy for RdCVFL (Table 4). The $\alpha$-values are relative weights compared with each other and, in our case, chosen to match the experimental data of the degeneration of $R_n$ and *C*. The values for $\alpha$ were chosen to be $\alpha _1=1$ and $\alpha _2=1.2$ to show the weights of control for rods and cones, respectively. Recall that the values for $\sigma _1$ is 192, which is $\delta _r R_{n0}$ rounded to the nearest whole number, and $\sigma _2$ is 234, $\delta _c C_0$ rounded to the nearest whole number. The lack of RdCVFL is illustrated by the $\delta _r$ and $\delta _c$ values being set equal to zero, and the light damage is reflected in the lowered values of $B_1$ and $B_2$. The numerical results show that with an $\epsilon$ value of 7.0e5 in (21), the numerical results agree with the experimental data; see Fig. 3. This shows that the progression of retinal diseases such as RP may be slowed using treatments that involve RdCVFL.

With the linearized control model (11)–(13), and using the same values for all the parameters and initial conditions, there is a higher saving rate but adjusting $\epsilon$ to be 1.8E+6, the savings rate is similar to the results of the nonlinear version.

**Table 3:** Parameters for $E_5$ stability; the estimated parameters result in $E_5$ stability at a reasonable level; see Wifvat et al. (2021)

Variable	Value	Units	Citation
$a_n$	1.25e$-$06	$\frac{1}{{\text{ day } \text{ mM }}}$	Estimated
$\beta _n$	2.0e$-$07	$\frac{1}{{\text{ day } \text{(rod } \text{ OS) }}}$	Estimated
$\gamma$	5.0e$-$5	$\frac{1}{{\text{ day } \text{(cone } \text{ OS) }}}$	Estimated
$\Gamma$	430.0	$\frac{1}{\text { day }}$	Estimated
$\kappa$	5.0e$-$3	$\frac{1}{{\text{ day } \text{ mM }}}$	Estimated
$\mu _n$	106.0	$\frac{{\text{ mM }}}{{\text{ day }}}$	Estimated
$\mu _c$	135.0	$\frac{{\text{ mM }}}{{\text{ day }}}$	Estimated
$K_{m}$	19	mM	Carruthers (2016)
$B_1$	1000	mM	Estimated
$B_2$	1200	mM	Estimated
$V_{\max }$	1728	$\frac{{\text{ mM }}}{{\text{ day }}}$	Carruthers (2016)
$\delta _n$	6.5e$-$5	mM	Léveillard et al. (2004)
$\delta _r$	1.0e$-$6	mM	Estimated
$\delta _c$	1.0e$-$4	mM	Estimated
$\nu _2$	7.65e$-$10	$\frac{1}{\text{ mM}^2}$	Estimated
p	1.0e$-$3	$\frac{\text{ mM }}{\text{ day }}$	Estimated

**Table 4:** Parameters changed for -/- LD; the estimated parameters give agreement with the degeneration observed in Elachouri et al. (2015)

Variable	Value	Units	Citation
$B_1$	700	mM	Estimated
$B_2$	840	mM	Estimated
$\delta _r$	0	mM	Elachouri et al. (2015)
$\delta _c$	0	mM	Elachouri et al. (2015)

**Fig. 4:**
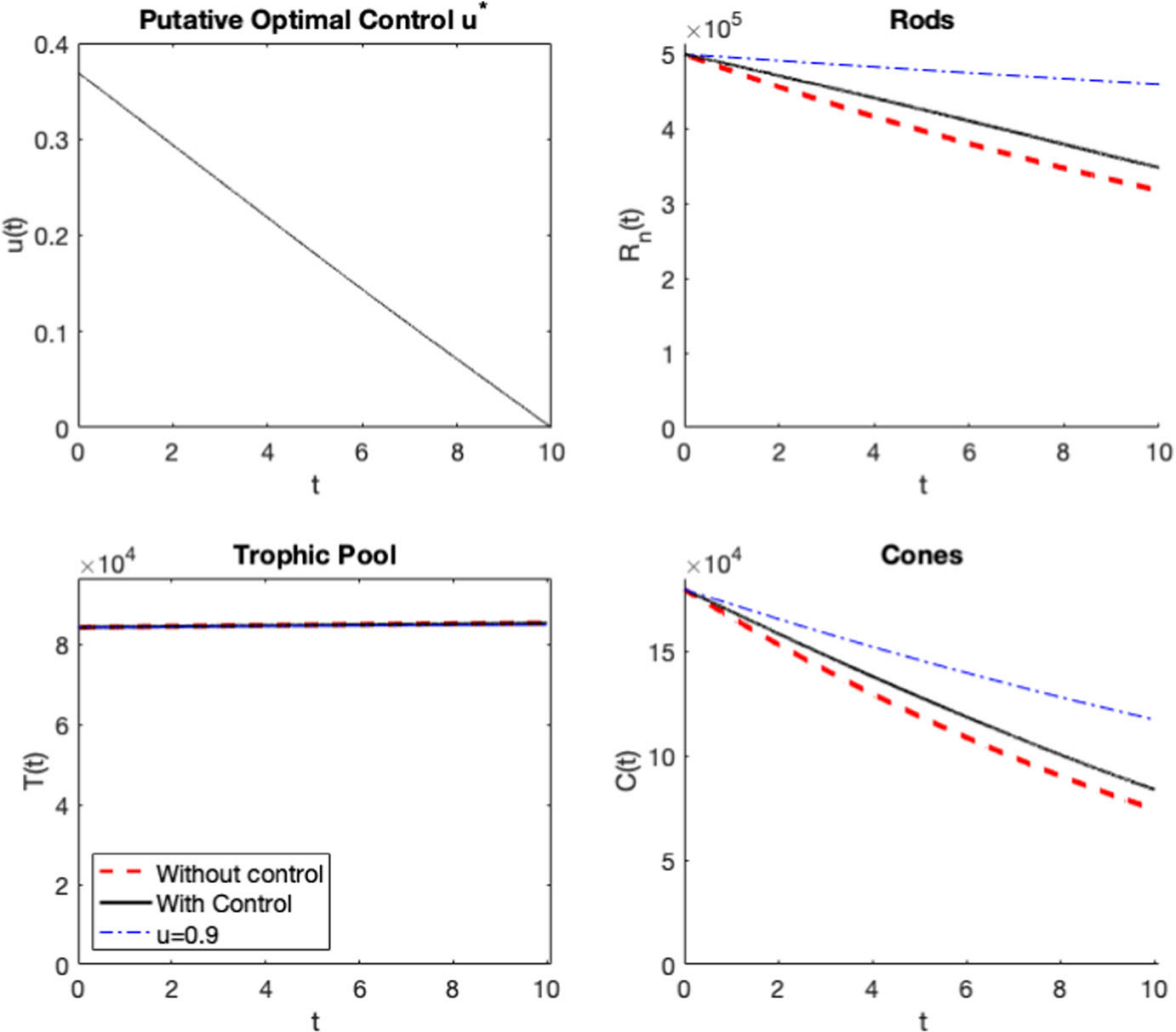
(Color figure online) The results of decay of both cones and rods, for the nonlinear RdCVFL control model (4)–(6). The initial conditions are $R_n= 0.5\text {e}6, C= 1.8\text {e}5, T=8\text {e}4$, a smaller value of the $R_n$ initial condition only, over the course of 10 days (reflected by the experimental results of Elachouri et al. (2015) for the *Nxnl1*-/- mouse), without control compared to the results of adding control to the rods and cones. Note that no experimental results are available for treatment with RdCVFL for these initial conditions (so there are no data points at $t=10$ as there were in Fig. 3)

## Sensitivity Analysis

**Fig. 5:**
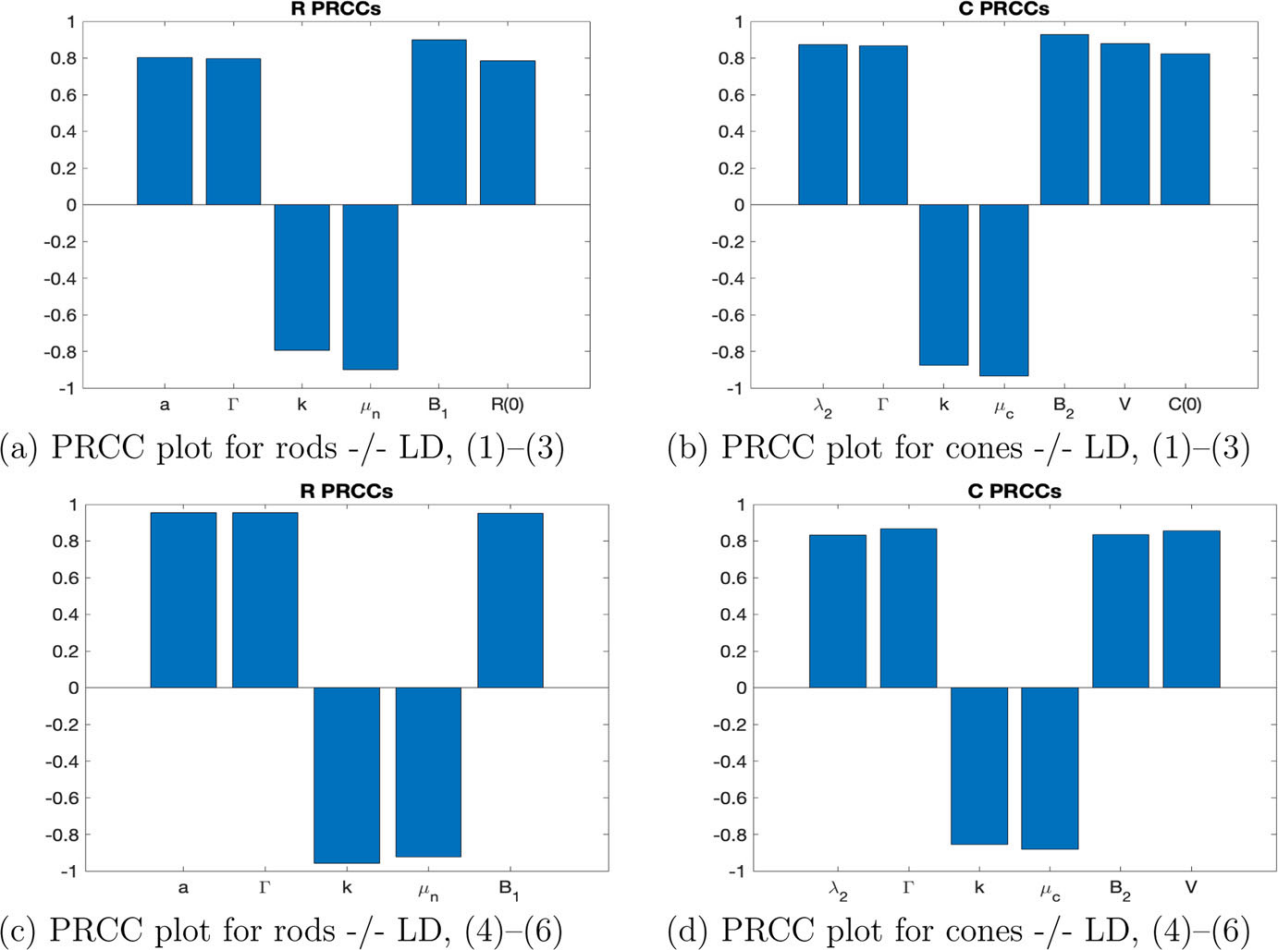
(Color figure online) **a, b** PRCC plots taken at 10 days for rods and cones, respectively, for the -/- mouse with LD for the model without control (1)–(3). The parameters are in Table 3 except the four changed parameters in Table 4. The **c, d** PRCC plots taken at 10 days for rods and cones, respectively, for the -/- mouse under the same conditions except now with nonlinear RdCVFL control *u* added (4)–(6). The difference is that the initial population of the state variable is no longer significant. Note that this is for the nonlinear model (4)–(6) with initial conditions $R_n= 6.4\text {e}6, C= 1.8\text {e}5, T=8\text {e}4$. See Fig. 3 for the control plot. The linearized control model (11)–(13) has the same qualitative results as shown in (**c, d**)

Next in order to see more clearly how the addition of the control impacts the rest of the model and the other parameters in the model, a sensitivity analysis is performed. The PRCC method is used in order to do a global sensitivity analysis and see which parameters have the strongest effects, and understand how variation in the parameter values affects the outcomes of our biological system. We first analyze the model without control (1)–(3), the nonlinear RdCVFL control model (4)–(6) and the linearized RdCVFL control model (11)–(13). Using the initial conditions presented in the control results in Fig. 3, $R_n= 6.4\text {e}6, C= 1.8\text {e}5, T=8\text {e}4$, we see from Fig. 5a–d the difference between the photoreceptor PRCC values without control (a)–(b), and with control (c)–(d). When there is not a control added to the model, the initial values of the state variables for rods and cones are important. However, when there is control added, these initial values are no longer significant. Notice that with or without control, the values $B_1$ and $B_2$ are significant. These parameters are correlated to the ROS, which RdCVFL has been experimentally shown to counteract. Similarly, the amount of RdCVFL present also affects shedding rates, which are represented by $\mu _n$ and $\mu _c$, which also show strong significance. Notice also that the quantities

38 $$\begin{aligned} D_T = \frac{\Gamma }{\kappa }, D_n = \frac{\mu _n}{a_n} \end{aligned}$$

are also significant, because the related parameters $\Gamma$, $\kappa$, $\mu _n$ and $a_n$ are all significant as well. These quantities are important because they have been shown in previous mathematical work (Camacho et al. 2016, 2016) to be key ratios that determine the stability of the equilibria. This shows that even in the case where RdCVFL is not present, key factors relating to energy uptake and the metabolic processes which are affected by RdCVFL are still significant.

**Fig. 6:**
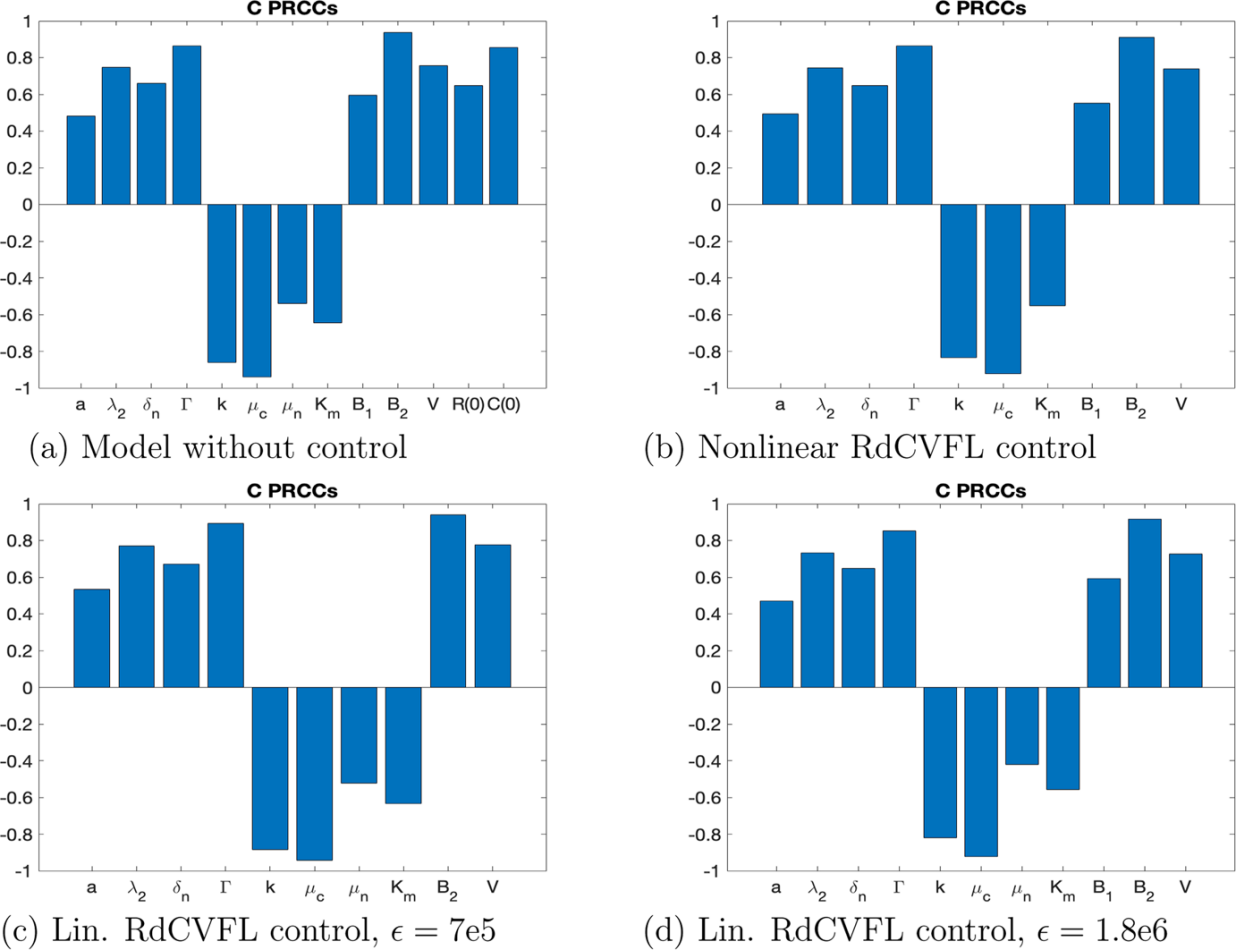
**a**–**d** PRCC plots taken at 10 days for cones, for the -/- mouse with LD and initial conditions $R_n= 0.5\text {e}6, C= 1.8\text {e}5, T=8\text {e}4$. The **a** is for the nonlinear model without control (1)–(3) and **b** is for the nonlinear control model (4)–(6). The **c, d** the PRCC plots for the linearized model with control (11)–(13) where **c** has the same $\epsilon$ value as the nonlinear model, $\epsilon =7\text {e}5$ and **d** has $\epsilon =1.8\text {e}6$. We observe that the qualitative results of the nonlinear and linearized models, **b, d** respectively, are similar and note that we considered 0.4 to be the value for statistical significance in the PRCC plots

For the initial values $R_n= 0.5\text {e}6, C= 1.8\text {e}5, T=8\text {e}4$, which are the initial conditions in the control model shown in Fig. 4, all of the PRCC plots are the same for both the nonlinear model without control (1)–(3), the nonlinear control model (4)–(6) and the linearized control model (11)–(13) except for four cases which are shown in Fig. 6. Only for the cones, we can see that several parameters are now more significant. For cones in the nonlinear model without control (1)–(3), the initial value of the rods is now significant, along with *a*, $\delta _n$, $\mu _n$, $K_m$ and $B_1$. Then for the nonlinear control model, the new significant parameters are *a*, $\delta _n$, $K_m$ and $B_1$. For the linearized control model, when $\epsilon =7\text {e}5$, which is the same $\epsilon$ that’s used for the nonlinear control model, the newly significant parameters are *a*, $\delta _n$, $\mu _n$ and $K_m$. Finally, when we use the $\epsilon$ value that is needed to get similar results from the linear control model as the nonlinear control model, $\epsilon =1.8e6$, we see the same significant parameters as for the previous $\epsilon$ value, except now $B_1$ is also significant as well. Additionally, the PRCC plots were also tested at the initial values $R_n= 3.6\text {e}6, C= 1.8\text {e}5, T=8\text {e}4$ and the results were the same as for the first initial values $R_n= 6.4\text {e}6, C= 1.8\text {e}5, T=8\text {e}4$ for both the rods and the cones.

## Conclusion

This is the first mathematical optimal control model of a light damaged retina and the potential effects of RdCVFL treatment. The RdCVFL control terms are nonlinear for biological accuracy but this results in the standard general theorems for existence of optimal controls failing to apply. We show the close agreement of the original model with the linearization of the control terms in the model and prove existence of an optimal control for the linearized model in Sect. 2. Because of this close agreement, we proceed under the assumption that a numerical optimal control obtained algorithmically and numerically may accurately represent an optimal control of the nonlinear system. The parameters are fit to real world experimental data for *Nxnl1*-/- mice, and the results show a savings rate for rods and cones that agrees with experiemental data. Similar results are obtained with the linear system by choosing a different $\epsilon$. We conclude that with the presence of RdCVFL added to the model of a diseased retina without any naturally occurring RdCVFL, the photoreceptor death is greatly reduced. In fact, it is potentially possible to retain the same amount of vision as a healthy eye, which is reflected in the control model reaching the same population levels as the experimental data of the nondiseased eye. This is an optimistic result for the further study of RdCVFL treatment in human trials.

Further, a global sensitivity and uncertainty analysis was performed to understand what the affect is of each input on the variability of the output of the model. This analysis of the control model was compared to the model without control to understand the differences which occur when control is added to the model. Without control, the PRCC plots show the significant parameters that affect the model output the most. These are the initial values *R*(0), *C*(0), the shedding rates and cell metabolism $\mu _n, \mu _c$, as well as other uptake and renewal factors such as $a, \Gamma$ and $\kappa$. The only difference when this analysis is performed on the optimal control model, is that the initial values *R*(0), *C*(0) are no longer significant. All models showed similar qualitative results for the listed initial conditions, meaning that it is especially important to look into these significant parameters. These results show that the potential RdCVFL treatment may still be able to take effect and help save the remaining photoreceptor population, even with a small initial population of photoreceptors. This is important because many individuals who suffer from RP do not attain a diagnosis or treatment until the disease has already progressed and affected their vision somewhat. Thus it is hopeful that for all patients, there can still be some saving effect for what vision is left.

Based on both the optimal control and the sensitivity and uncertainty analysis, RdCVFL is shown to be an important factor for the survival of photoreceptor populations. In retinas that do not have RdCVFL being produced, a treatment involving RdCVFL may be able to help patients retain vision and slow the progression of the disease. Based on the sensitivity and uncertainty results, this type of potential treatment shows promise for helping at any stage of such retinal diseases. Overall, RdCVFL has been shown to be an important factor in helping the retinas maintain photoreceptor survival and may potentially help patients retain vision.

## Positivity and Boundedness of Solutions

### Existence of Unique Global Solutions for the System without Control

#### Theorem 6

There exist locally unique solutions to the initial value problems of the system of equations defined in (1)–(3) for ($R(0)=R_{n_0}$, $C(0)=C_0$, $T(0)=T_0$) in ${\mathbb {R}}^3$ where $R_{n0}\ne \frac{-B_1}{\delta _r }$ and $C_0\ne \frac{-B_2}{\delta _c}$. The coordinate planes and hence the positive octant $\Omega = \{(R,C,T):R>0, C>0, T>0\}$ are invariant under the local flow. Moreover, all solutions starting in ${\bar{\Omega }} = \{(R,C,T):R\ge 0, C\ge 0, T\ge 0\}$ are bounded forward in time and hence are defined for $[0,\infty )$.

#### Proof

The right-hand side of the system (1)–(3) are rational functions of $R_n, C$ and *T* and are continuously differentiable and locally Lipschitz continuous. Thus, by the Picard Lindelöf theorem (Coddington and Levinson 1955 Theorem 3.1), these initial value problems have local unique solutions. Clearly, if $R_n=0$, then $\dot{R_n}=0$, and so on. Thus solutions starting on the coordinate planes stay on the coordinate planes. Hence, by uniqueness, solutions cannot cross the coordinate planes and the positive open and closed octants $\Omega$ and ${\bar{\Omega }}$ are invariant. Note that because all parameters are non-negative and ${\bar{\Omega }}$ includes non-negative values, the conditions $R_{n0}\ne \frac{-B_1}{\delta _r }$ and $C_0\ne \frac{-B_2}{\delta _c}$ hold. To show that solutions in ${\bar{\Omega }}$ stay bounded for all positive times, consider the following.

Let *h*, *j*, *n*,  and *q* be defined as the following:

$$\begin{aligned}{} & {} h:=\max \{a_n, \nu _2 (V_{\max }+p) \} \\{} & {} n:=\min \left\{ \frac{\mu _n}{B1}, \frac{\mu _c}{B2} \right\} \\{} & {} j:=\min \{ \beta , \gamma \} \\{} & {} q:=\frac{\Gamma +n}{2\kappa } \end{aligned}$$

Note that *h*, *j*, *n*,  and *q* are all positive since the parameters in the respective definitions are positive. Let $W=R+C+(\frac{h}{j})T$. Then, $W\ge 0$, and we have the following equations:

$$\begin{aligned} \begin{aligned} \frac{dW}{dt}= \frac{dR}{dt}+\frac{dC}{dt}+\left( \frac{h}{j}\right) \frac{dT}{dt}, \end{aligned} \end{aligned}$$

which turns into the following after substituting the equation definitions:

$$\begin{aligned}{} & {} = R Ta_n-\mu _n \left( \frac{ R}{B_1 + \delta _r R}\right) +\nu _2\left( \frac{V_{\max }(\delta _nR)^2}{K_{mg}^2+(\delta _nR)^2}+p \right) CT-\mu _c \left( \frac{ C}{B_2 + \delta _c C}\right) \\{} & {} \quad +\left( \frac{h}{j}\right) T(\Gamma -\kappa T-\beta R-\gamma C). \end{aligned}$$

Then, expanding and rearranging the terms, we get:

$$\begin{aligned}{} & {} \le a_nRT + \left( \nu _2 \frac{V_{\max }(\delta _nR)^2}{K_{mg}^2+(\delta _nR)^2}+\nu _2 p\right) CT-\mu _n \left( \frac{ R}{B_1 + \delta _r R}\right) -\mu _c \left( \frac{C}{B_2 + \delta _c C}\right) \\{} & {} \quad -\beta \left( \frac{h}{j}\right) RT -\gamma \left( \frac{h}{j}\right) CT+\left( \frac{h}{j}\right) T (\Gamma -\kappa T). \end{aligned}$$

Note that

39 $$\begin{aligned} \frac{V_{\max }(\delta _nR)^2}{K_{mg}^2+(\delta _nR)^2}\le V_{\max }, \ \frac{ R}{B_1 +\delta _r R}\le \frac{1}{B_1} R, \ \text {and} \ \frac{ C}{B_2+\delta _c C}\le \frac{1}{B_2} C. \end{aligned}$$

Then, we have the following inequality:

$$\begin{aligned}{} & {} \frac{dW}{dt} \le a_nRT + \nu _2\left( V_{\max }+p\right) CT-\mu _n \left( \frac{1}{B_1 }\right) R -\mu _c \left( \frac{1}{B_2}\right) C \\{} & {} \quad -\beta \left( \frac{h}{j}\right) RT -\gamma \left( \frac{h}{j}\right) CT+\left( \frac{h}{j}\right) T (\Gamma -\kappa T). \end{aligned}$$

Then, substituting *h*, *n*, *j* and *q* as defined previously, we get the following:

$$\begin{aligned} \frac{dW}{dt} \le hRT + hCT - nR -nC-j \left( \frac{h}{j}\right) RT -j\left( \frac{h}{j}\right) CT+\left( \frac{h}{j}\right) T (\Gamma -\kappa T) \end{aligned}$$

Which reduces to:

$$\begin{aligned} \frac{dW}{dt} = -n(R+C)+\left( \frac{h}{j}\right) T(\Gamma -\kappa T) \end{aligned}$$

Finally, substituting back $W=R+C+(\frac{h}{j})T$, this turns into:

40 $$\begin{aligned} \frac{dW}{dt}= & {} -n(W-\left( \frac{h}{j}\right) T)+\left( \frac{h}{j}\right) T(\Gamma -\kappa T)\nonumber \\= & {} -n W+\left( \frac{h}{j}\right) T+\left( \frac{h}{j}\right) T(\Gamma -\kappa T)\nonumber \\\le & {} -nW+q. \end{aligned}$$

Then, $\frac{dW}{dt}\le -nW +q$, and we get

41 $$\begin{aligned} W(t)\le \frac{q}{n}+\left( W(0)-\frac{q}{n} \right) e^{-nt}. \end{aligned}$$

Therefore, W is bounded along solutions for positive times starting in ${\bar{\Omega }}$. Thus, $R_n, C$ and *T* are also bounded on ${\bar{\Omega }}$ for positive times. Since $\Omega$ is invariant, and solutions starting in ${\bar{\Omega }}$ stay bounded for positive times, the solutions exist for all positive times. $\square$

As a direct consequence of (40) we have the following corollary which we will use when arguing the existence of optimal controls.

#### Corollary 1

Using the notation and parameters as in Theorem 6, the compact *simplex*

42 $$\begin{aligned} S'=\left\{ (R,C,T)\in [0,\infty ]^3 :W=R+C+\left( \frac{h}{j}\right) T\le \frac{q}{n}+1 \right\} \end{aligned}$$

is positively forward invariant under the flow of the system (1)–(3).

#### Proof

From equation (40) it immediately follows that if $W>\frac{q}{n}$ then $\frac{dW}{dt}<0$. $\square$

#### Remark 1

The statements of Theorem 6 and Corollary 1 remain true if the system (1)–(3) is replaced by the system (4)–(6), or by system (11)–(13). Indeed, in the proof of Theorem 6 above, the second and third estimates in the inequalities (39) remain valid upon replacing $\delta _r R\ge 0$ and $\delta _c C\ge 0$ by $\sigma _1 u\ge 0$ and $\sigma _2 u\ge 0$, respectively.
